# A sweet corn metabolic regulatory network spanning the entire life cycle

**DOI:** 10.1186/s43897-025-00201-y

**Published:** 2026-03-03

**Authors:** Lili Ma, Jinhua Zuo, Alisdair R. Fernie, Shiyu Liu, Xinyuan Zhou, Zixia Jing, Yunxiang Wang, Xinyi Feng, Yiting Ren, Caie Wu, Ronghuan Wang, Yaxing Shi, Yanyan Zheng

**Affiliations:** 1 Institute of Agri-food Processing and Nutrition, Beijing Academy of Agriculture and Forestry Sciences, Beijing Key Laboratory of Fruits and VegeTable Storage and Processing, Key Laboratory of Vegetable Postharvest Processing, Ministry of Agriculture and Rural Affairs, Beijing, 100097 China; 2 College of Light Industry and Food Engineering, Nanjing Forestry University, Nanjing, 210037 Jiangsu China; 3 Max Planck Institute of Molecular Plant Physiology, 14476 Potsdam Golm, Germany; 4 Beijing Technology and Business University (BTBU), Beijing Engineering and Technology Research Center of Food Additives, Beijing Advanced Innovation Center for Food Nutrition and Human Health, School of Food and Health, Beijing, 100048 China; 5 Institute of Maize. Beijing Academy of Agriculture and Forestry Sciences, Beijing, 100097 China

Sweet corn’s growth is regulated by genetic, hormonal and environmental interactions (Haque et al. 2019). Although maize gene regulatory networks (GRNs) have revealed key regulators (Huang et al. 2018; Zhou et al. 2020; Zhu et al. 2023), multi-omics studies throughout sweet corn life cycle remain limited. We developed the Sweet Corn Metabolic Network (SCMN) dataset using the ‘Nongkenuo 336’ variety. Samples included fruits at nine stages (0, 4, 8, 12, 16, 20, 24, 28, and 32 days after silking (DAS)), roots, stems, and leaves at V10 (tenth leaf), VT (tasseling) and 28 DAS, and flowers at VT and R1 (silking) (Fig. 1A). The SCMN provides a spatiotemporal transcriptomic-metabolomic atlas for sweet corn research.

**Fig. 1:**
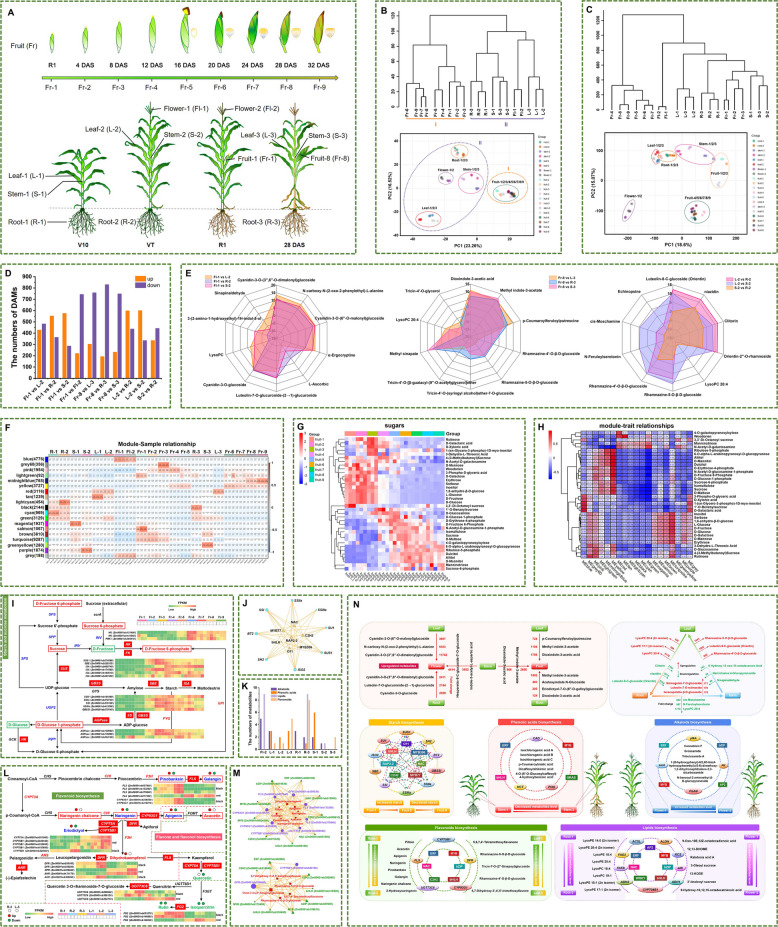
The spatiotemporal transcriptome-metabolome landscape of sweet corn across its growth cycle. **A** Nine developmental stages of fruit and four stages of roots, stems, leaves and flowers for sweet corn. **B** Cluster dendrogram and PCA of metabolome data from the 20 tissues samples. **C** Cluster dendrogram and PCA of transcriptome data from the 20 tissues samples. **D** The numbers of DAMs in 10 pairwise comparisons. **E** Radar chart illustrating the top 10 metabolites with the most significant changes in pairwise tissue comparisons, including flower vs. other tissues, fruit vs. other tissues, and specific contrasts between leaf-2, stem-2, and root-2. **F** Module-sample correlation plot. Each row represents a module, and each column represents a sample. **G** Cluster heatmap of sugars in metabolites. **H** Heatmap showing module-trait correlations. Each column corresponds to a module indicated by different colors. Each row corresponds to a sugar compound. Each block contains the corresponding correlation coefficient between the module and trait. **I** Starch and sucrose metabolism pathway. The genes filled in red represent the DEGs identified in the yellow module. The red and green metabolites represent positive and negative correlations with the yellow module, respectively. **J** Putative transcriptional regulatory network related to starch synthesis in the yellow module.** K** Number of major metabolite categories with specific high accumulation in each tissue. **L** Flavonoid biosynthesis pathway and flavone and flavonol biosynthesis pathway. The genes filled in red represent the DEGs identified in the black or red module. Metabolites with red borders represent specific high accumulation flavonoids identified in root-3. The red and green metabolites indicate an increase and decrease in abundance, respectively. The blue metabolites indicate an increase in root-3 and decrease in leaf-3 in abundance. **M** Putative transcriptional regulatory networks associated with flavonoids synthesis in the black and red modules, respectively. **N** The regulatory model of metabolite tissue- and stage-specific accumulation in sweet corn across its growth cycle. The red and green arrow represent an increase and decrease in metabolite levels, respectively. Ellipses represent genes, rectangles represent transcription factors. The dashed arrow pointing from the transcription factor to the gene indicates that the transcription factor may regulate the expression of the structural gene

Metabolomic profiling revealed 1,629 metabolic features exhibiting distinct accumulation patterns across diverse tissues (Table S1, Fig. S1). These metabolic features clustered into two distinct groups: those predominantly present in fruits and those enriched in other tissues (Fig. 1B), demonstrating tissue-specific metabolic specialization during sweet corn development. Pairwise comparisons identified differentially accumulated metabolites (DAMs) between tissues (Fig. 1D). In the comparison of flower, root, stem, and leaf, flowers accumulated high levels of the pigments cyanidin-3-*O*-(3'',6''-*O*-dimalonyl) glucoside and cyanidin-3-*O*-(6''-*O*-malonyl) glucoside (Fig. 1E), contributing to their blue or purple coloration (Zhou et al. 2024). Fruits exhibited significantly higher levels of dioxindole-3-acetic acid and methyl indole-3-acetate compared to roots, stems, and leaves (Fig. 1E). In the comparison of root, stem, and leaf samples the orientin, rhamnazine-5-*O*-β-D-glucoside, and rhamnazine-4'-*O*-β-D-glucoside accumulated substantially in the leaves, while roots displayed higher LysoPC 20:4 levels (Fig. 1E).

In total, 57,296 genes were identified as expressed in at least one sample (Table S2), while samples from the same tissues clustered closely together (Fig. 1C). Notably, fruits at early developmental stages (fruits-1/2/3) formed two distinct categories separate from fruits at later stages (Fig. 1C). These results underscore the tissue-specific gene expression and metabolite accumulation during sweet corn development.

To further elucidate the regulatory mechanisms underlying metabolic changes, we conducted weighted gene co-expression network analysis (WGCNA) and identified 18 co-expression modules (Fig. 1F, S2). Sugars in fruits at different stages revealed two distinct clusters (Fig. 1G). The yellow module positively correlated with sugars accumulation, such as D-glucose-1-phosphate, D-fructose-6-phosphate, D-lactose, D-maltose (Fig. 1H). The gene expression pattern within the yellow module is shown in Fig. S3. A total of 19 starch synthesis-related genes were identified within yellow module (Fig. 1I), and 98 TFs showed strong correlations with 16 starch synthesis-related genes (Table S3). These TFs may serve as potential regulators of starch synthesis and formed a correlation network (Fig. 1J). Notably, the ethylene-responsive transcription factor RAP2-2 was the most connected key regulator in the network (Table S3).

We identified 83 stage-specific metabolites (FC > 3) in sweet corn tissues (Table S4). Flavonoids (17), phenolic acids (15), lipids (19) and alkaloids (16) were the most abundant compound classes to which these metabolites belonged (Fig. 1K; Table S4). Our study focused on the specifically highly accumulated metabolites: flavonoids in roots and leaves, phenolic acids in stems, alkaloids in flowers, and lipids in roots and flowers (Table S4).

Eight and five distinct specifically highly accumulated flavonoids (SHAFs) were detected in root-3 and leaf-3, respectively (Table S4). The black and red modules displayed positive correlation with root-3 and leaf-3, respectively (Fig. 1F), and were also positively correlated with 25 and 130 flavonoids, respectively (Table S5). To investigate the regulation of flavonoid biosynthesis, we identified 13 and nine key genes in the black and red modules, respectively, including amongst others*bifunctional dihydroflavonol 4-reductase/flavanone 4-reductases* (*DFR*), *flavonoid 3'-monooxygenase* (*CYP75B1*), *anthocyanidin reductase* (*ANR*) (Table S6, S7). Most genes displayed significantly higher expression in root-3 or leaf-3 (Fig. 1L, S4). Among them, eight and two genes showed strong positive correlation with SHAFs in root-3 and leaf-3, respecively (Table S6, S7). Additionally, 186 and 160 TFs were identified within the black and red module, respectively (Table S8). Strong positive correlations (CC > 0.8) were observed among 61 TFs, eight key genes and eight SHAFs in root-3, as well as among 30 TFs, two key genes, and five SHAFs in leaf-3 (Table S6, S7). Given that these TFs formed a correlation network with structural genes and flavonoids (Fig. 1M), they may be potential regulators of SHAFs in root-3 and leaf-3 (Feng et al. 2023; Han et al. 2023).

Six phenolic acids exhibited particularly high accumulation in stem-1 (Table S4). The magenta module showed the strongest positive correlation with stem-1 and 17 phenolic acid (Fig. 1F; Table S5). We identified ten phenolic acid-related genes involved in phenylpropanoid biosynthesis within magenta module, including *shikimate O-hydroxycinnamoyltransferases* (*HCT*), *peroxidase* (*POD*), *cinnamyl-alcohol dehydrogenase* (*CAD*)(Table S9). These genes exhibited significantly higher expression levels in stem-1 (Fig. S5). A strong positive correlation between six phenolic acid-related genes (*CAD*, 2*HCTs*, 3*PODs*) and the six specifically highly accumulated phenolic acids (SHAPAs) in stem-1 was also observed (Table S9). 136 TFs were identified in the magenta module (Table S8), of which 97 exhibited strong positive correlations with six key genes and six SHAPAs (Table S9). Given that these TFs formed a correlation network with structural genes and phenolic acids (Fig. S5), we postulate their role in regulating SHAPAs in stem-1 (Sun et al. 2019).

Five alkaloids with specifically high accumulation were detected in flower-2 (Table S4). The blue module showed the strongest positive correlation with flower-2 and 34 alkaloids (Fig. 1F; Table S5). Since plant alkaloids are typically synthesized from amino acids (Alibi et al. 2021), we studied amino acid biosynthesis and identified 22 alkaloid-related genes in blue module, including *pyruvate kinase* (*PK*), *6-phosphofructokinase 1* (*pfkA*) and *2,3-bisphosphoglycerate-dependent phosphoglycerate mutase* (*PGAM*) (Table S10). Most genes showed higher expression in flower tissues (Fig. S6), and four genes strongly correlated with four specifically highly accumulated alkaloids (SHAAs) in flower-2 (Table S10). Within the blue module, 149 TFs were identified (Table S8). A strong positive correlation was observed among 35 TFs, four alkaloid-related genes, and four specifically highly accumulated alkaloids (Table S10). These TFs from the AP2/ERF, bZIP, and MYB families are thus potential regulators of the SHAAs in flower-2 (Deng et al. 2018), and as would be anticipated formed a common correlation network (Fig. S6).

Nine lipids showed tissue-specific high accumulation in root-3, while another nine were prominent in flower-2 (Table S4). The black and blue modules showed the strongest correlation with root-3 and flower-2, respectively (Fig. 1F), and were positively correlated with 47 and 37 lipids, respectively (Table S5). In both the black and blue modules, we examined 13 lipid-related genes involved in fatty acid biosynthesis, degradation, and metabolism, including the key genes *3-ketoacyl-CoA synthase* (*KCS*), *aldehyde dehydrogenase (NAD*^+^*)* (*ALDH*) and *alcohol dehydrogenase* (*ADH*) (Table S11, S12). Most genes were significantly higher expressed in root-3 or flower (Fig. S7). Nine and four genes showed positive correlation with specifically high-accumulation lipids (SHALs) in root-3 and flower-2, respectively (Table S11, S12). The strong positive correlations were observed among 108 TFs, nine key genes and eight SHALs in root-3, as well as among 35 TFs, four key genes, and seven SHALs in flower-2 (Table S11, S12). These TFs are potential candidate regulators of SHALs and as would therefore be anticipated formed a common correlation network (Fig. S7).

In summary, this study uncovers tissue- and stage-specific dynamics of gene expression and metabolites in sweet corn, we propose a comprehensive model to elucidate the regulatory mechanisms of tissue- and stage-specific accumulation of metabolites such as starch, flavonoids, phenolic acids, alkaloids, and lipids throughout the entire growth cycle of sweet corn (Fig. 1N). These findings contribute valuable insights for improving sweet corn quality.

## Supplementary Information

Supplementary Material 1. Table S1. The 1629 metabolites identified across various tissues. Table S2. The 57296 genes identified in various tissue samples. Table S3. The starch synthesis-related genes and transcription factors in the yellow module. Table S4. The metabolites with stage-specific high accumulation in roots, stems, leaves and flowers tissues of sweet corn. Table S5. Metabolites highly correlated with the module. Table S6. The flavonoid-related genes, TFs, and their correlation, as well as their correlation with specific high accumulation flavonoids in the black module. Table S7. The flavonoid-related genes, TFs, and their correlation, as well as their correlation with specific high accumulation flavonoids in the red module. Table S8. The transcription factors (TFs) within the modules. Table S9. The phenolic acid-related genes, TFs, and their correlation, as well as their correlation with specific high accumulation phenolic acids in the magenta module. Table S10. The alkaloid-related genes, TFs, and their correlation, as well as their correlation with specific high accumulation alkaloids in the blue module. Table S11. The lipid-related genes, TFs, and their correlation, as well as their correlation with specific high accumulation lipids in the black module. Table S12. The lipid-related genes, TFs, and their correlation, as well as their correlation with specific high accumulation lipids in the blue module. 

Supplementary Material 2. Figure S1. Metabolite clustering heatmap in various tissues of sweet corn. Figure S2. Hierarchical clustering dendrogram shows co-expression modules that are color-coded. Figure S3. The expression profiles of genes in the yellow module. Figure S4. Analysis of flavonoids with stage- and tissue-specific high accumulation. Figure S5. Analysis of specifically highly accumulated phenolic acids in stem-1. Figure S6. Analysis of specifically highly accumulated alkaloids in flower-2. Figure S7. Analysis of specifically highly accumulated lipids in root-3 and flower-2. Materials and Methods.

## Data Availability

The datasets used and/or analysed during the current study are available from the corresponding author on reasonable request.
