# Supplementary material for: A sweet corn metabolic regulatory network spanning the entire life cycle
Source: Mol Hortic. 2026 Mar 3;6:18. doi: 10.1186/s43897-025-00201-y (PMC12954910; doi:10.1186/s43897-025-00201-y)
Supplement: Supplementary file 2 — Supplementary Material 2. Figure S1. Metabolite clustering heatmap in various tissues of sweet corn. Figure S2. Hierarchical clustering dendrogram shows co-expression modules that are color-coded. Figure S3. The expression profiles of genes in the yellow module. Figure S4. Analysis of flavonoids with stage- and tissue-specific high accumulation. Figure S5. Analysis of specifically highly accumulated phenolic acids in stem-1. Figure S6. Analysis of specifically highly accumulated alkaloids in flower-2. Figure S7. Analysis of specifically highly accumulated lipids in root-3 and flower-2. Materials and Methods. [file 43897_2025_201_MOESM2_ESM.docx]

**Supplementary Material 2**

**
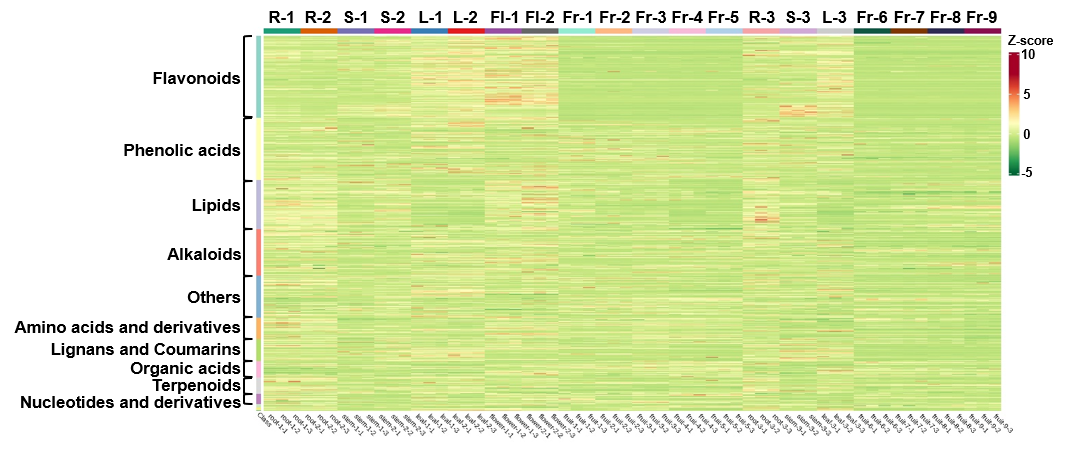
**

**Fig. S1.** Metabolite clustering heatmap in various tissues of sweet corn.


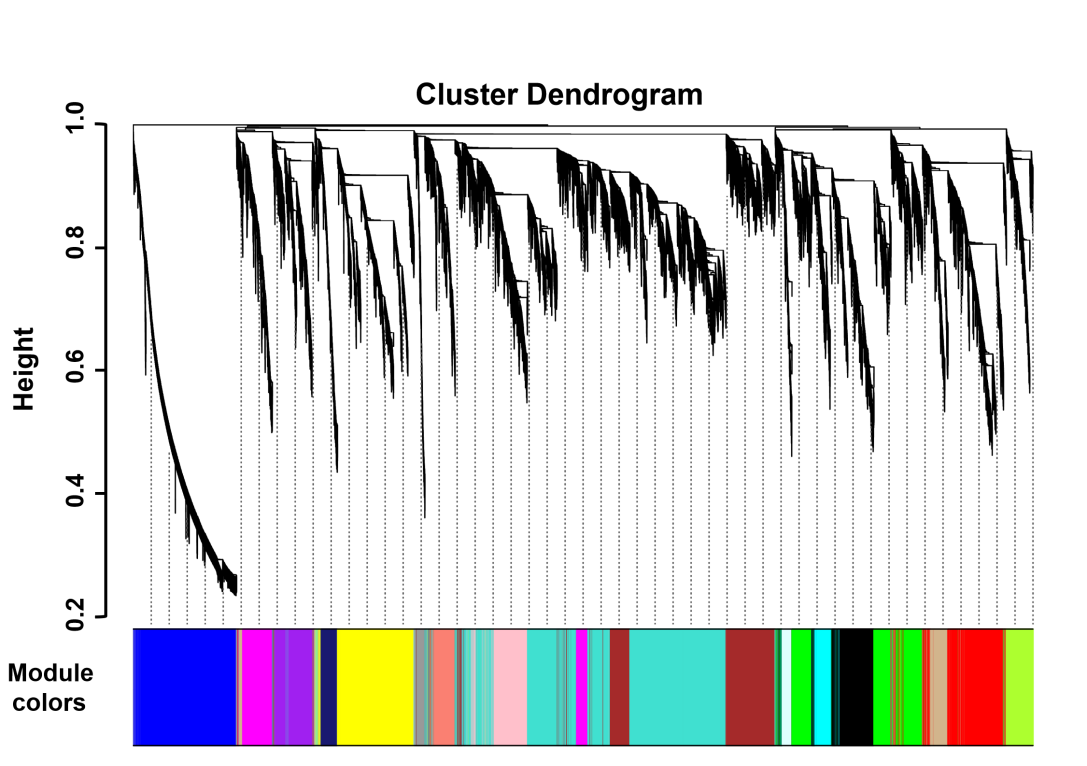


**Fig. S2.** Hierarchical clustering dendrogram shows co-expression modules that are color-coded. The major branches constitute 18 modules labeled by different colors (grey module is for genes unassigned).


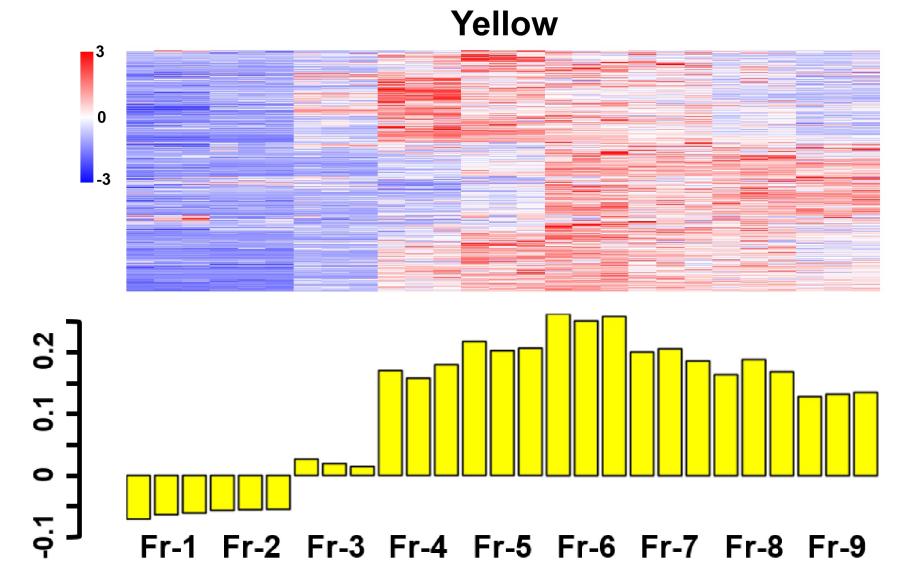


**Fig. S3.** The expression profiles of genes in the yellow module. The upper part is the clustering heatmap of genes within the module, with red indicating high expression and blue indicating low expression. The lower part shows the module eigengene expression patterns in fruit samples at different stages.


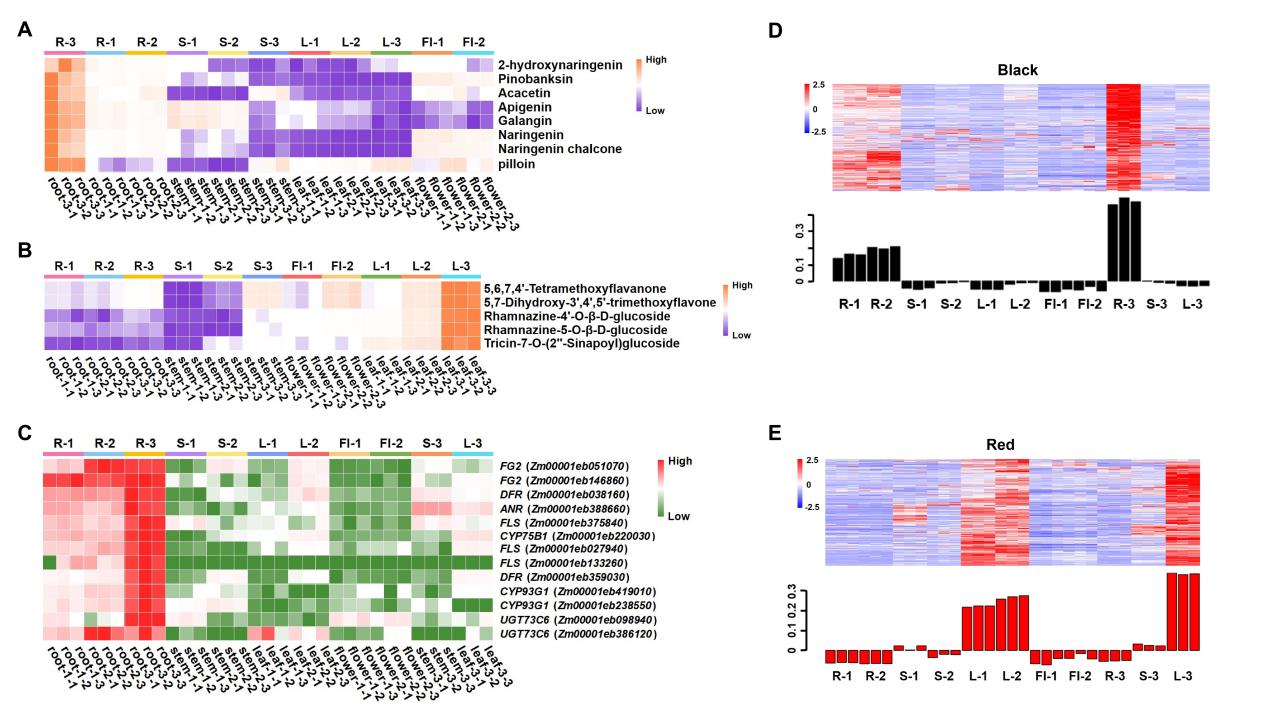


**Fig. S4.** Analysis of flavonoids with stage- and tissue-specific high accumulation. **(A)-(B)** Heatmap of flavonoids with specific high accumulation in root-3 and leaf-3. **(C)** Heat map of genes related to flavonoid synthesis in the black module. **(D)-(E)** The expression profiles of genes in the black and red modules. The upper part is the clustering heatmap of genes within the module, with red indicating high expression and blue indicating low expression. The lower part shows the module eigengene expression patterns across various tissues.


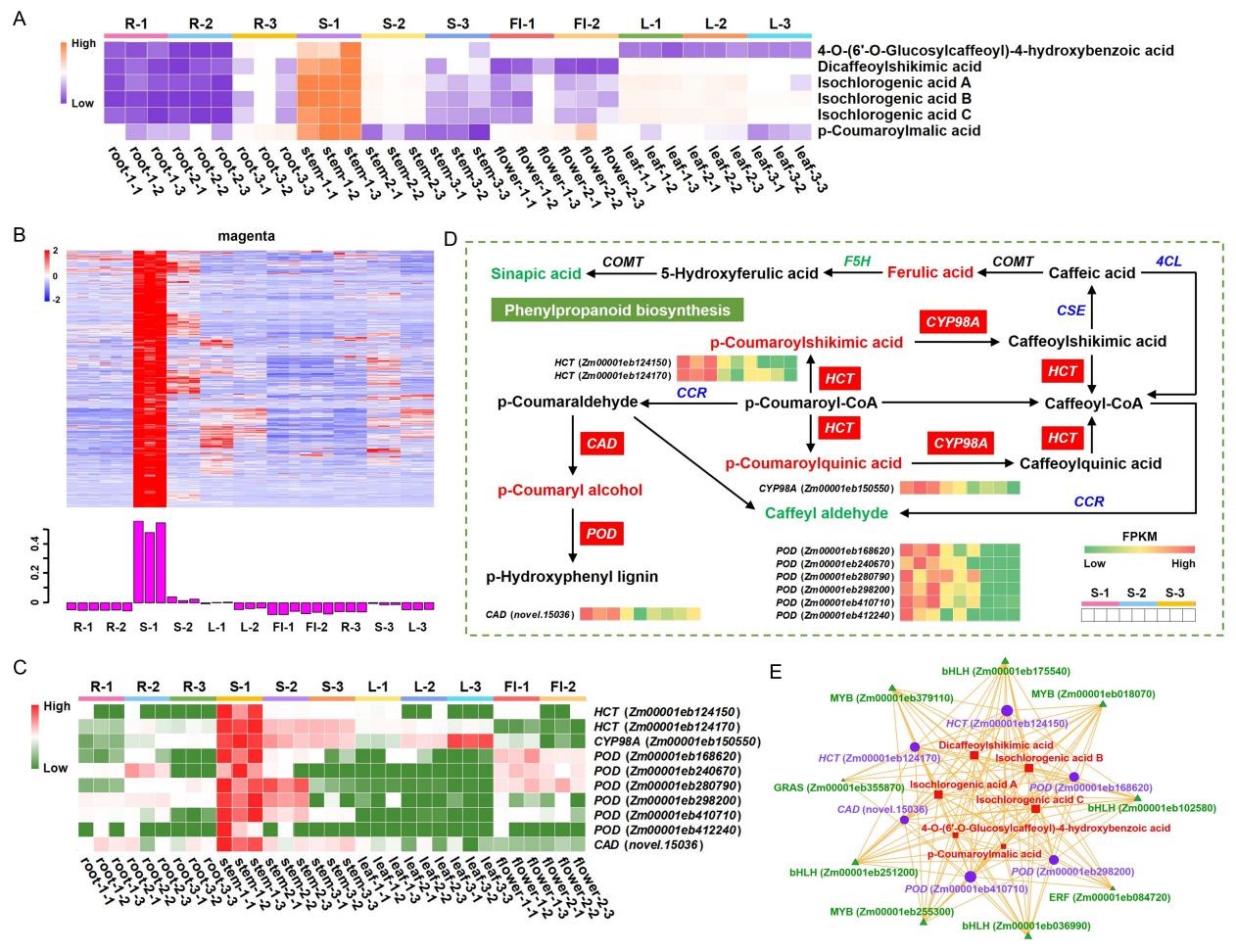


**Fig. S5.** Analysis of specifically highly accumulated phenolic acids in stem-1. **(A)** Heatmap of phenolic acids with specific high accumulation in stem-1. **(B)** The expression profiles of genes in the magenta module. The upper part is the clustering heatmap of genes within the module, with red indicating high expression and blue indicating low expression. The lower part shows the module eigengene expression patterns across various tissues. **(C)** Heat map of genes related to phenolic acids synthesis in the magenta module. **(D)** Phenylpropanoid biosynthesis pathway. The genes filled in red represent the DEGs identified in the magenta module. The green genes represent downregulated DEGs in stem-1, the blue genes represent both upregulated and downregulated DEGs in stem-1 identified in other modules. The red and green metabolites indicate an increase and decrease in abundance in stem-1, respectively. COMT, caffeic acid 3-*O*-methyltransferase/acetylserotonin O-methyltransferase; F5H, ferulate-5-hydroxylase; 4CL, 4-coumarate-CoA ligase; CSE, caffeoylshikimate esterase; HCT, shikimate *O*-hydroxycinnamoyltransferase; CYP98A, 5-*O*-(4-coumaroyl)-D-quinate 3'-monooxygenase; CCR, cinnamoyl-CoA reductase; CAD, cinnamyl-alcohol dehydrogenase; POD, peroxidase. **(E)** Putative transcriptional regulatory network related to phenolic acids synthesis in the magenta module.


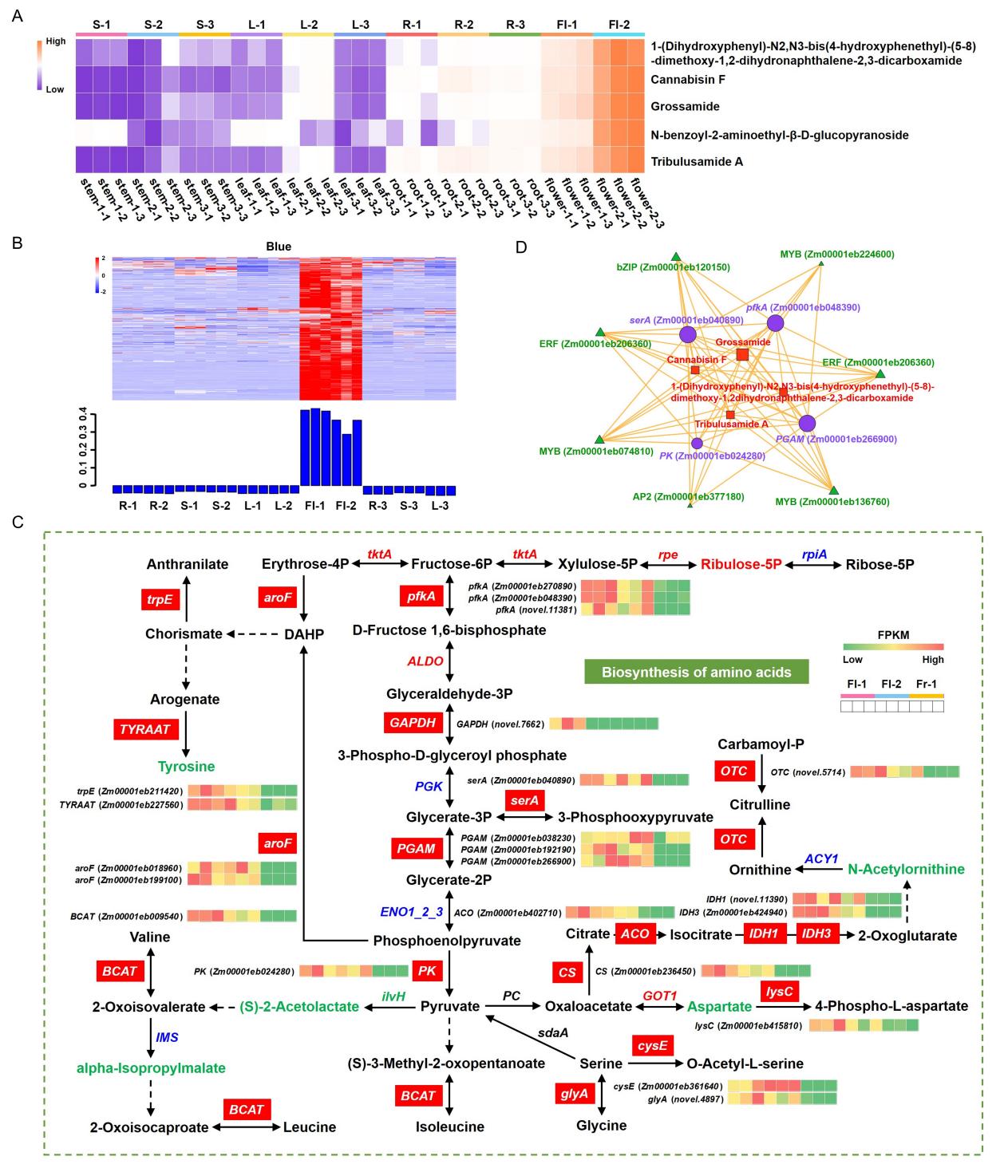


**Fig. S6.** Analysis of specifically highly accumulated alkaloids in flower-2. **(A)** Heatmap of alkaloids with specific high accumulation in flower-2. **(B)** The expression profiles of genes in the blue module. The upper part is the clustering heatmap of genes within the module, with red indicating high expression and blue indicating low expression. The lower part shows the module eigengene expression patterns across various tissues. **(C)** Biosynthesis of amino acids pathway. The genes filled in red represent the DEGs identified in the blue module. The red and green genes represent upregulated and downregulated DEGs in flower-2, respectively, the blue genes represent both upregulated and downregulated DEGs in flower-2 identified in other modules. The red and green metabolites indicate an increase and decrease in abundance in flower-2 compared to fruit-1, respectively. tktA, transketolase; rpe, ribulose-phosphate 3-epimerase; rpiA, ribose 5-phosphate isomerase A; trpE, anthranilate synthase component I; aroF, 3-deoxy-7-phosphoheptulonate synthase; pfkA, 6-phosphofructokinase 1; ALDO, fructose-bisphosphate aldolase, class I; TYRAAT, arogenate dehydrogenase (NADP^+^); GAPDH, glyceraldehyde 3-phosphate dehydrogenase (phosphorylating); PGK, phosphoglycerate kinase; serA, D-3-phosphoglycerate dehydrogenase/2-oxoglutarate reductase; PGAM, 2,3-bisphosphoglycerate-dependent phosphoglycerate mutase; ENO1_2_3, enolase 1/2/3; PK, pyruvate kinase; sdaA, L-serine dehydratase; cysE, serine O-acetyltransferase; glyA, glycine hydroxymethyltransferase; ilvH, acetolactate synthase I/III small subunit; BCAT, branched-chain amino acid aminotransferase; IMS, 2-isopropylmalate synthase; PC, pyruvate carboxylase; CS, citrate synthase; ACO, aconitate hydratase; IDH1, isocitrate dehydrogenase; IDH3, isocitrate dehydrogenase (NAD^+^); ACY1, aminoacylase; OTC, ornithine carbamoyltransferase; GOT1, aspartate aminotransferase; lysC, aspartate kinase. **(D)** Putative transcriptional regulatory network related to alkaloids synthesis in the blue module.


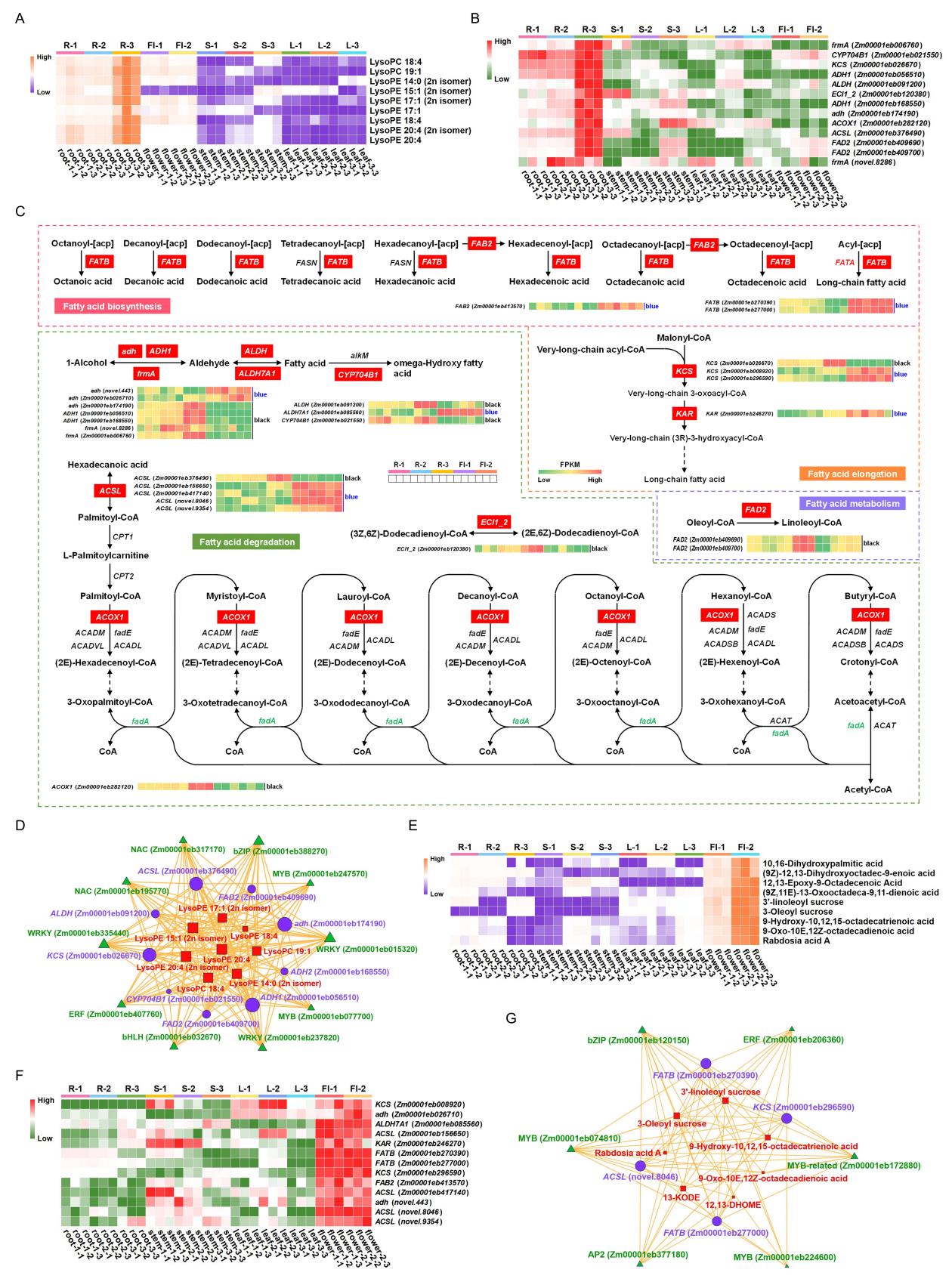


**Fig. S7.** Analysis of specifically highly accumulated lipids in root-3 and flower-2. **(A)** Heatmap of lipids with specific high accumulation in root-3. **(B)** Heat map of genes related to lipids synthesis in the black module. **(C) Fatty acid biosynthesis, degradation, elongation, and metabolism pathways.** The genes filled in red represent the DEGs identified in the black or blue module. FATB, fatty acyl-ACP thioesterase B; FATA, fatty acyl-ACP thioesterase A; FAB2, acyl-[acyl-carrier protein] desaturase; adh, alcohol dehydrogenase; ADH1, alcohol dehydrogenase class-P; frmA, S-(hydroxymethyl)glutathione dehydrogenase/alcohol dehydrogenase; ALDH, aldehyde dehydrogenase (NAD^+^); ALDH7A1, aldehyde dehydrogenase family 7 member A1; alkM, alkane 1-monooxygenase; CYP704B1, long-chain fatty acid omega-monooxygenase; ACSL, long-chain acyl-CoA synthetase; CPT1, carnitine *O*-palmitoyltransferase 1; CPT2, carnitine *O*-palmitoyltransferase 2; ACOX1, acyl-CoA oxidase; ACADM, acyl-CoA dehydrogenase; fadE, acyl-CoA dehydrogenase; ACADVL, very long chain acyl-CoA dehydrogenase; ACADL, long-chain-acyl-CoA dehydrogenase; fadA, acetyl-CoA acyltransferase; ACADS, butyryl-CoA dehydrogenase; ACADSB, short-chain 2-methylacyl-CoA dehydrogenase; ACAT, acetyl-CoA C-acetyltransferase; ECI1_2, Delta3-Delta2-enoyl-CoA isomerase; KCS, 3-ketoacyl-CoA synthase; KAR, 17beta-estradiol 17-dehydrogenase/very-long-chain 3-oxoacyl-CoA reductase; FAD2, omega-6 fatty acid desaturase/acyl-lipid omega-6 desaturase. **(D)** Putative transcriptional regulatory network related to lipids synthesis in the black module. **(E)** Heatmap of lipids with specific high accumulation in flower-2. **(F)** Heat map of genes related to lipids synthesis in the blue module. **(G)** Putative transcriptional regulatory network related to lipids synthesis in the blue module.

**Materials and methods**

**Plant materials**

The fresh corn (*Zea mays* L.) cultivar ‘Nongkenuo 336’, a white sweet-waxy-compound corn variety, were cultivated at the “Jingxianyuan” base in Henan Zhai Town, Miyun District, Beijing, China. The growth and developmental stages of fresh corn were categorized into vegetative (V) and reproductive (R) stages. **Root, stem, and leaf tissues** were collected at the **V10 (tenth leaf)**, **VT (tasseling)**, and **28 days after silking (DAS)** stages, labeled as root-1, root-2, root-3; stem-1, stem-2, stem-3; and leaf-1, leaf-2, leaf-3, respectively. **Flower tissues** were collected at the **VT** and **R1 (silking)** stages and recorded as flower-1 and flower-2, respectively. **Fruit tissues** were collected across nine different stages, from **R1 to R1+32 d** (0, 4, 8, 12, 16, 20, 24, 28, 32 DAS), and recorded as fruit-1, fruit-2, fruit-3, fruit-4, fruit-5, fruit-6, fruit-7, fruit-8, and fruit-9, respectively. All tissue samples were cut into small pieces, promptly frozen in liquid nitrogen, and stored at **-80 °C**. Samples from ten individual plants were pooled to form a single biological replicate, and three biological replicates were used for subsequent transcriptome and metabolome analyses.

**Metabolite extraction and analysis**

Biological samples were freeze-dried using a lyophilizer (Scientz-100F) and ground into powder (30 Hz, 1.5 min) using a grinder (MM 400, Retsch). A 50 mg aliquot of powdered sample was weighed using an electronic balance (MS105DM) and extracted with 1,200 µL of pre-cooled (at **-20 °C**) 70% methanolic aqueous solution containing an internal standard. For smaller samples (<50 mg), the extraction solvent was adjusted proportionally. The mixture was vortexed for 30 seconds every 30 minutes, repeated six times. After centrifugation (12,000 rpm, 3 minutes), the supernatant was collected, filtered through a **0.22 µm microporous membrane**, and stored in injection vials for **UPLC-MS/MS analysis**.

**Principal Component Analysis (PCA)** was performed using the prcomp function in **R** ([www.r-project.org](http://www.r-project.org)) on unit variance-scaled data. **Hierarchical Cluster Analysis (HCA)** and Pearson Correlation Coefficients (PCC) between samples were calculated using the ComplexHeatmap R package. For HCA, normalized signal intensities of metabolites (unit variance scaling) were visualized as a color spectrum. Differentially accumulated metabolites (DAMs) were identified using **VIP > 1** (derived from OPLS-DA) and **|Log2FC| ≥ 1.0** thresholds. **OPLS-DA** (Orthogonal Partial Least Squares Discriminant Analysis) was conducted using the MetaboAnalystR package, with data log2-transformed and mean-centered. Permutation tests (200 permutations) were performed to prevent overfitting. Identified metabolites were annotated using the **KEGG Compound** database ([http://www.kegg.jp/kegg/compound/](http://www.kegg.jp/kegg/compound/)) and mapped to the **KEGG Pathway** database ([http://www.kegg.jp/kegg/pathway.html](http://www.kegg.jp/kegg/pathway.html)). Enrichment analysis of pathways was performed using **Metabolite Set Enrichment Analysis (MSEA)**, with significance determined using the hypergeometric test (p-values).

**Transcriptome sequencing and analysis**

Transcriptome sequencing and analysis were performed following previously described method (Bai et al. 2025). The reference genome and annotation files for Zea mays were downloaded from the Ensembl database ([https://ftp.ensemblgenomes.ebi.ac.uk/pub/plants/release-55/fasta/zea_mays/dna/](https://ftp.ensemblgenomes.ebi.ac.uk/pub/plants/release-55/fasta/zea_mays/dna/)). **HISAT** was used to construct the genome index and map clean reads to the reference genome. **StringTie** was employed for new gene prediction, and **featureCounts** was used to calculate gene alignments. Gene expression levels were quantified as **FPKM** (Fragments Per Kilobase of transcript per Million mapped reads). **DESeq2** was used to identify differentially expressed genes (DEGs) between groups, with p-values corrected using the Benjamini-Hochberg method. Thresholds for significant differential expression were set at corrected p-value < 0.05 and **|log2foldchange| ≥ 1.0**. The enrichment analysis is performed based on the hypergeometric test. For **KEGG**, pathways were analyzed using hypergeometric distribution tests. For **GO**, enrichment was conducted based on Gene Ontology terms. Weighted gene co-expression network analysis (**WGCNA**) was performed to identify co-expression modules and explore correlations between gene expression and phenotypic traits.
